# Initial Surgical Strategy for the Treatment of Type A Acute Aortic Dissection: Does Proximal or Distal Extension of the Aortic Resection Influence the Outcomes?

**DOI:** 10.3390/ijerph19148878

**Published:** 2022-07-21

**Authors:** Carlo Bassano, Marta Pugliese, Charles Mve Mvondo, Calogera Pisano, Paolo Nardi, Dario Buioni, Fabio Bertoldo, Mattia Scognamiglio, Alessandro C. Salvati, Claudia Altieri, Giovanni Ruvolo

**Affiliations:** 1 Cardiac Surgery Unit, Department of Surgery, Tor Vergata University, 00133 Rome, Italy; lindapisano82@gmail.com (C.P.); pa.nardi4@libero.it (P.N.); dario.buioni@ptvonline.it (D.B.); fabio.bertoldo@uniroma2.it (F.B.); mattia.scognamiglio@ptvonline.it (M.S.); alessandrocristian.salvati@ptvonline.it (A.C.S.); giovanni.ruvolo@uniroma2.it (G.R.); 2 Cardiac Surgery Unit, Sant’Anna Hospital, 88100 Catanzaro, Italy; marta.pugliese@gmail.com (M.P.); mmvondocarlo@yahoo.fr (C.M.M.); 3 Cardiology Unit of the Cardiac Surgery Division, Tor Vergata University Polyclinic, Viale Oxford 81, 00133 Rome, Italy; claudia.altieri@ptvonline.it

**Keywords:** acute aortic dissection, aortic arch, surgical treatment

## Abstract

(1) Background: We sought to analyze and compare the outcomes in terms of early and late mortality and freedom from a redo operation in patients undergoing surgical treatment for a type A acute aortic dissection in relation to the initial surgical treatment strategy, i.e., proximal or distal extension of the aortic segment resection, compared with isolated resection of the supracoronary ascending aorta. (2) Methods: This is a retrospective study in which we included 269 patients who underwent operations for a type A acute aortic dissection in the Department of Cardiac Surgery of Tor Vergata University from May 2006 to May 2016. The patients were grouped according to the extent of the performed surgical treatment: isolated replacement of the supracoronary ascending aorta (NE, no extension), replacement of the aortic root (PE, proximal extension), replacement of the aortic arch (DE, distal extension), and both (BE, bilateral extension). The analyzed variables were in-hospital mortality, postoperative complications (incidence of neurological damage, renal failure and need for prolonged intubation), late mortality and need for a redo operation. (3) Results: Unilateral cerebral perfusion was performed in 49.3% of the patients, and bilateral perfusion—in 50.6%. The overall in-hospital mortality was 31.97%. In the multivariate analysis, advanced age, cardiopulmonary bypass time and preoperative orotracheal intubation were independent predictors of in-hospital mortality. In the population of patients who survived the surgery, the probability of survival at 92 months was 70 ± 5%, the probability of freedom from a redo operation was 71.5 ± 5%, the probability of freedom from the combined end-point death and a redo operation was 50 ± 5%. The re-intervention rate in the general population was 16.9%. The overall probability of freedom from re-intervention was higher in patients undergoing aortic root replacement, although not reaching a level of statistical significance. Patients who underwent aortic arch treatment showed reduced survival. (4) Conclusions: In the treatment of type A acute aortic dissection, all the surgical strategies adopted were associated with satisfactory long-term survival. In the group of patients in which the aortic root had not been replaced, we observed reduced event-free survival.

## 1. Introduction

Type A acute aortic dissection is a disease with a catastrophic impact on the patient’s life, and emergency surgery is the only possible and reasonable treatment at an early stage. Its incidence is around 30 cases/million/year, and the natural history of the disease is burdened by an untreated mortality rate of 58% according to the International Registry of Acute Aortic Dissections (IRAD) [1], with the risk of death increasing by 1% per hour in the first 48 h from the onset of symptoms [2,3]. Despite numerous diagnostic innovations and the development of new surgical techniques and advances in postoperative medical treatment, it is still a disease with a high mortality rate (15–30%) even when it has undergone surgery. Prompt surgical treatment is the only therapeutic option that can improve the otherwise poor prognosis of this disease. For 60-year-old patients discharged from the hospital after surgical aortic repair, the survival rate is as high as 96% at 1 year and 93% at 3 years [4,5]. Although the patient’s preoperative status and age are important and unchangeable risk factors affecting in-hospital mortality, the different surgical techniques used can also influence the postoperative course and long-term outcome of these patients [6]. Indeed, several surgical aspects in the management of this condition still need to be clarified better in order to refine the selection criteria for each technique and decide the extent of a replacement. Among the various open questions in this field, the management of the aortic arch and root is one of the most debated issues. The primary goal of surgery is patient’s survival. Accordingly, the first emergency surgery is usually focused on proximal ascending aorta replacement in order to prevent aortic rupture and cardiac tamponade, correcting acute aortic insufficiency, preventing dissection of the coronary ostium and ensuring patency of the supra-aortic trunks. Resection of the intimal entry laceration is indeed a well-established principle to reduce morbidity and mortality. However, limiting the treatment to the most threatening lesions without worrying about the fate of aortic segments with residual disease seems unjustified because of the high risk of future aortic complications. On the other hand, the rate of the need for a redo operation varies between 15 and 30% at 10 years, although most studies in the literature do not always specify whether the dilatation involves the aortic root or the distal aorta [7]. This aspect has prompted more and more surgeons to opt for a more aggressive initial surgical strategy. The complete resection of all entry sites would be necessary to achieve complete thrombosis of the false lumen and to avoid the risk of malperfusion and/or of the aneurysmal expansion during follow-up, these last two dreaded events. Unfortunately, the initial surgery is almost never able to achieve a radical treatment of the entire disease, especially in patients with a localized intimal tear in the descending thoracic and abdominal aorta, and in those with re-entry ruptures extending throughout the aorta wall. In recent years, the emergence of hybrid techniques combining conventional surgery with endovascular procedures have opened up new perspectives for the treatment of these patients [8]. The need for increasingly complex procedures has consequently led to improved techniques for cerebral protection and prevention of organ damage. There is still no consensus in the literature on the first-choice strategy for the treatment of an acute aortic dissection, which is an extremely complex condition with a wide range of clinical presentations and anatomical variants.

The aim of our study was to analyze and compare early and late outcomes, especially freedom from late re-intervention, in patients undergoing surgical treatment for a type A acute aortic dissection (AAD) in relation to the initial surgical strategy, i.e., proximal or distal extension of the aortic resection in comparison with the isolated resection of the supracoronary ascending aorta.

## 2. Materials and Methods

### 2.1. Patients’ Population

Our study population consisted of 269 patients who underwent operations for type A acute aortic dissection (AAD) at the Cardiac Surgery Unit of the Surgical Department of the Tor Vergata, University of Rome from May 2006 to May 2016. The mean age of patients was 63 ± 12.7 years (range 16–87 years), with male predominance (191 patients, 71%). Forty-eight patients (18%) were aged > 75 years at the time of surgery. Forty patients (15%) arrived intubated in the operating room, and sixteen (6%) had undergone previous cardiac surgery. The demographic and preoperative characteristics of the patients are summarized in Table 1, Table 2 and Table 3. The study was conducted according to the guidelines of the Declaration of Helsinki and approved by the Independent Ethics Committee of the Tor Vergata University Polyclinic (N. ethical code 46/22). All patients gave their informed surgical consent.

### 2.2. Patient Selection and Study Design

As a referral center for cardiac surgical emergencies, we performed a retrospective study on an “all comers” population to obtain a reliable picture of real-world results, without exclusion of patients for any reason. We intentionally limited our research to a pre-“hybrid prosthesis” era, since many centers are not yet equipped with these later devices or lack the knowledge to implant them. Furthermore, frozen elephant trunk procedures were not routinely performed during the surgical period analyzed in our study. Pre-, intra- and postoperative data were retrospectively collected through analysis of medical records. All patients underwent a preoperative CT scan with a contrast medium and trans-esophageal echocardiographic evaluation. A complete assessment of the diameter of the aortic root and the ascending aorta and the site of the intimal lesion was always performed. Trans-esophageal echocardiography allowed a specific assessment of aortic valve morphology and any associated degree of insufficiency or other concomitant disease. At the time of dissection, the diameter of the ascending aorta was 54.2 ± 10.4 mm measured by means of a CT scan and/or trans-esophageal echocardiographic exams. The patients were grouped according with the extent of surgical treatment received: 96 (35.7%) underwent replacement of the ascending aorta alone in its supracoronary segment (NE, no extension), 41 (15.2%)—replacement of the entire ascending aorta segment including the aortic root (PE, proximal extension), 112 (41.6%)—replacement of the aortic arch (DE, distal extension), and 20 (7.4%)—both (BE, bilateral extension).

### 2.3. Surgical Techniques

Surgery was performed by median longitudinal sternotomy in all patients. After systemic heparinization, cardiopulmonary bypass was established via axillary (49.8% of patients), femoral (41.6% of patients) or central arterial cannulation (8.6% of patients), while venous drainage was achieved via right atrial, bi-caval or femoral venous cannulation, depending on the specific circumstances and the surgeon’s preference. Myocardial protection was achieved with the use of different cardioplegia solutions [9], although more often with a selective injection of Custudiol^®^ cold crystalloid cardioplegic solution into the coronary ostia. Left ventricular drainage was performed through the right superior pulmonary vein. All surgical procedures were conducted under moderate or deep hypothermia, based on the expected systemic circulatory arrest time. In the case of aortic arch surgery, once the desired temperature was reached, systemic circulatory arrest was initiated, and cerebral protection was provided by selective hypothermic unilateral (50.6% of patients) or bilateral (49.3%) cerebral perfusion. Monolateral cerebral perfusion was maintained by using an arterial cannula inserted into the axillary artery and proximal clamping of the origin of the anonymous arterial trunk. Bilateral central perfusion, on the other hand, was performed according with the Kazui technique: both the anonymous truncus arteriosus and the left common carotid artery were cannulated using dedicated cannulas and perfused using an independent pump at a flow rate of 10–15 mL/kg/min. In 31 patients, a bilateral cerebral perfusion technique was developed in our center without the use of additional cannulas: it involved cannulation of the right axillary artery and tangential clamping of the aortic arch convexity. In other cases, a modified Kazui technique was employed: when the right axillary artery was used for the arterial return, and side-clamping of the aortic arch was judged unsafe, only the left common carotid artery was selectively cannulated through the aortic lumen, the right one was indirectly perfused via the right axillary artery itself. The decision whether or not to perform a concomitant aortic root and/or aortic arch replacement and aortic valve procedure was the responsibility of the operating surgeon, based on the site of the intimal rupture, the size of the aortic root and arch, the degree of aortic valve regurgitation and the patient’s clinical conditions. Proximal surgery was generally completed during the cooling phase, prior to circulatory arrest. Distal repair was performed using the open technique under hypothermia. Upon completion of the distal anastomosis, cardiopulmonary bypass was restored, and rewarming started. The cannulation sites and intraoperative characteristics are shown in Table 4, Table 5 and Table 6. Preoperative monitoring included Swan-Ganz catheter placement, double arterial cannulation for continuous monitoring blood pressure (radial and femoral), measurement of body temperature, assessment of cerebral oxygenation using spectroscopy (INVOS^®^ System, Somanetics Corp., Troy, MI, USA) and, more recently, transcranial Doppler.

### 2.4. Follow-Up

The mean duration of follow-up was 39.5 ± 37 months (median 37 range 0 to 132 months). The sum of the actual months of observation was 10,635 months per patient out of a theoretical maximum follow-up of 11,572 months per patient, indicating that the completeness of follow-up was 92%. At the follow-up, each patient underwent echocardiography and thoraco-abdominal CT-scan angiography. In patients unable to be evaluated in our department, a telephone interview was conducted. For patients who died, a telephone interview with the relatives was performed.

### 2.5. Analyzed Variables

The variables analyzed were in-hospital mortality, postoperative complications, i.e., incidence of neurological damage, renal failure and need for prolonged intubation, late mortality, overall freedom from aorta-related events and need for late re-intervention.

### 2.6. Statistics

Statistical analysis was performed using the Stat View 4.5 system (SAS Institute Inc., Abacus Concepts, Berkeley, CA, USA). Continuous variables were expressed as the mean value ± standard deviation, categorical variables were expressed as the absolute value and percentage rate. The comparison of continuous variables was carried out by means of *t*-tests for unpaired data. For categorical variables, contingency tables, the chi-squared and Fisher’s exact tests were used. Late survival and freedom from late re-intervention were calculated using Kaplan–Maier curves. Actuarial curves were compared using the Mantel–Cox log-rank test. All variables with a 0.1 *p*-value at the univariate analysis were included in the multivariable analysis using the Cox regression model. All the variables with a *p*-value less than 0.05 were considered statistically significant.

## 3. Results

### 3.1. Intraoperative Data

Intraoperative characteristics are shown in Table 4 and Table 5. Unilateral cerebral perfusion was performed in 49.3% of patients, bilateral perfusion—in 50.6%. The overall in-hospital mortality was 31.97%, or 86 patients out of 269.

In detail, operative mortality in the four groups of patients, i.e., NE (no extension), PE (proximal extension), DE (distal extension), BE (bilateral extension) groups of patients, and postoperative complications are reported in Table 6, Table 7, Table 8 and Table 9.

The variables significantly associated with operative mortality at the univariate analysis were complete aortic arch replacement, longer cross clamping and cardiopulmonary bypass times, age greater than 75 years, prolonged ventilation, acute renal failure, preoperative orotracheal intubation (OTI) and preoperative neurological damage. Including all these variables in the multivariate analysis, advanced age, cardiopulmonary bypass time and preoperative OTI were independent predictors of in-hospital mortality (Table 10). The mortality rate for patients > 75 years was 52% in comparison with 27% for younger patients (*p* = 0.018). Previous cardiac surgery, associated procedures on the aortic valve, myocardial revascularization, and time and mode of cerebral perfusion were not identified as risk factors in neither linear regression nor logistic regression analysis. There were 23 deaths in the operating room due to hemodynamic deficit, heart failure or uncontrollable hemorrhage.

### 3.2. Long-Term Results

During the follow-up, there were 33 deaths. Of them, four cases were related to re-intervention or re-dissection, four cases were due to hemorrhage, eleven cases due to malignancy, two cases due to heart failure and twelve cases due to unknown cause.

**Figure 1:**
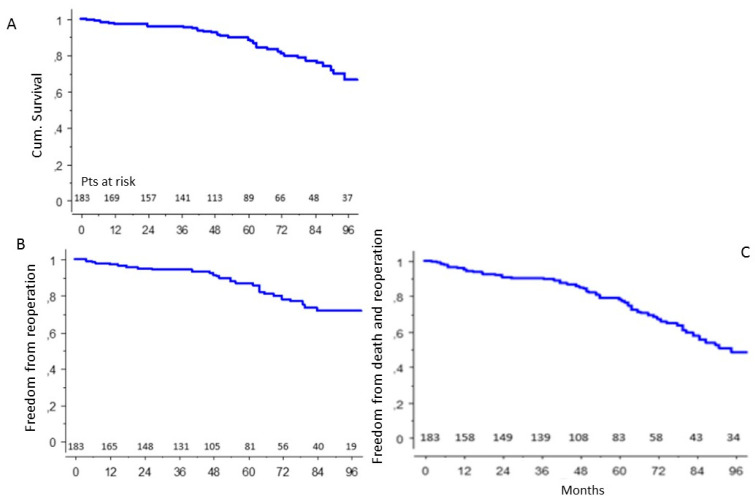
Cumulative survival (**A**), freedom from reoperation (**B**), freedom from composite end-point (death + reoperation) (**C**) in the general population of patients.

In the population of patients who survived surgery, the probability of survival at 92 months was 70 ± 5% (Figure 1A), the probability of freedom from a redo operation was 71.5 ± 5% (Figure 1B), the probability of freedom from the combined end-point death and a redo operation was 50 ± 5% (Figure 1C).

The re-intervention rate in the general population was 16.9%; the relative rates of the four subgroups of patients are reported in Table 11.

### 3.3. NE Patients Group vs. Extended Intervention (Proximal, Distal or Both) Groups of Patients

**Figure 2:**
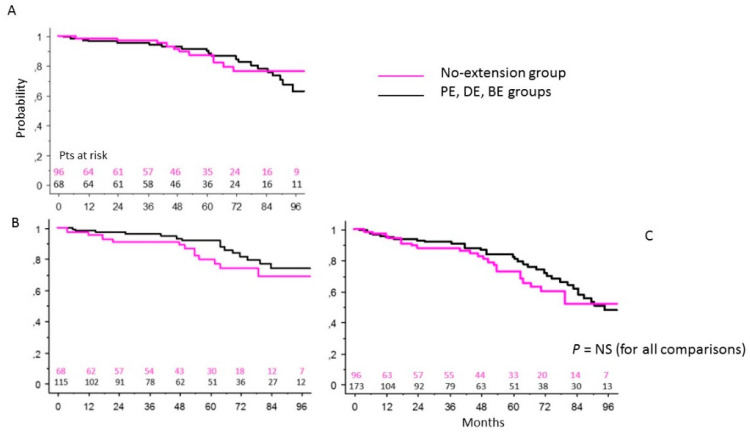
Survival (**A**), freedom from reoperation (**B**), freedom from composite end-point (death + reoperation) (**C**) in the NE group of patients in comparison with other groups of patients (log-rank test).

At 92 months, in the NE group of patients, the survival was 76 ± 6% vs. 67 ± 7% in patients with proximal and/or distal extension of the surgical treatment (*p* = 0.86) (Figure 2A); the freedom from a redo operation was 74 ± 6% vs. 68 ± 8% (*p* = 0.36) (Figure 2B), and the freedom from death and a redo operation 52 ± 8% vs. 51 ± 7% (*p* = 0.4) (Figure 2C). The overall re-intervention rate was 20.3%. Using the chi-square analysis, we found that the actual incidence rate of reoperation on the aortic root was significantly higher in the NE group compared to those in the other (PE, DE, BE) patient groups (13 vs. 1.7%; *p* = 0.0025).

### 3.4. PE Group vs. Patients’ Population without Aortic Root Surgery

**Figure 3:**
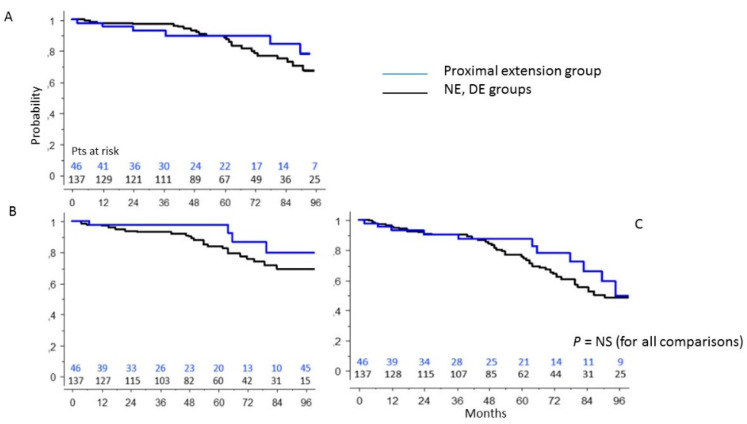
Survival (**A**), freedom from reoperation (**B**), freedom from composite end-point (death + reoperation) (**C**) in the PE group of patients in comparison with those in the NE and DE groups of patients (log-rank test).

Patients undergoing proximal extension compared with patients without aortic root treatment had a 92-month survival rate of 76 ± 10% vs. 68 ± 6% (*p* = 0.68) (Figure 3A), a freedom from re-intervention of 79 ± 10% vs. 69 ± 6% (*p* =0.16) (Figure 3B), and a freedom from combined re-intervention- and-death of 59 ± 11% vs. 48 ± 6% (*p* = 0.31) (Figure 3C). The overall re-intervention rate was 8.7%. Using chi-square analysis, we found that the actual incidence rate of late reoperation on the aortic root was significantly lower in PE and BE groups of patients in comparison with that in NE and only DE groups (0 vs. 8%; *p* = 0.04).

### 3.5. DE Group vs. Non-Arch Surgery Patients’ Population

**Figure 4:**
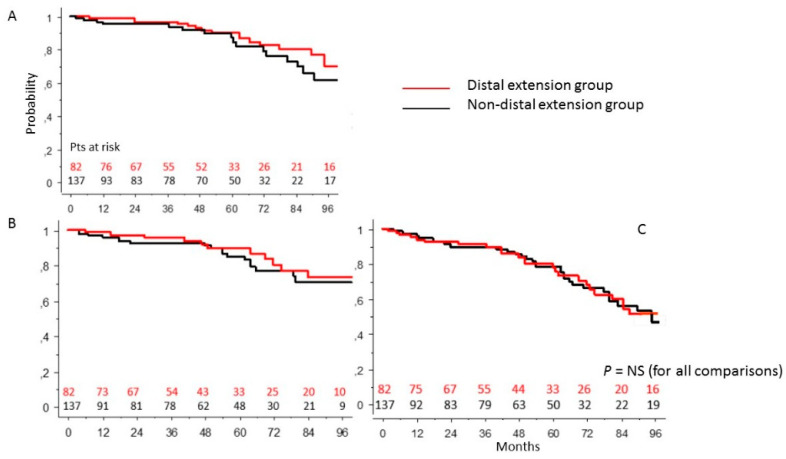
Survival (**A**), freedom from reoperation (**B**), freedom from composite end-point (death + reoperation) (**C**) in the DE group of patients in comparison with those in the non-distal extension group of patients (log-rank test).

Patients who underwent more or less conservative treatment of the aortic arch had, in comparison with patients who did not undergo distal extension, a probability of survival at 92 months of 68.2 ± 8% vs. 64 ± 8% (*p* = 0.53) (Figure 4A), freedom from re-intervention of 73 ± 7.5% vs. 70 ± 6.5% (*p* = 0.8) (Figure 4B), and freedom from re-intervention and death of 53 ± 8% vs. 48 ± 7% (*p* = 0.95) (Figure 4C). The overall re-intervention rate was 12.6%. The actual incidence rate of late reoperation on the proximal segment of the ascending aorta was significantly lower in the DE group of patients in comparison with that in the NE group (2.9 vs. 13.2%; *p* = 0.03).

### 3.6. DE Group: Conservative Surgery vs. Radical Surgery of the Aortic Arch

**Figure 5:**
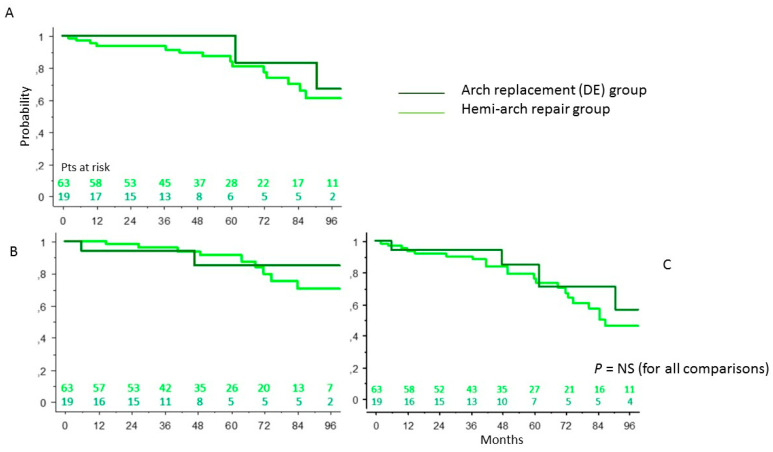
Survival (**A**), freedom from reoperation (**B**), freedom from composite end-point (death + reoperation) (**C**) in patients undergoing total aortic arch replacement vs. patients undergoing hemi-arch repair (log-rank test).

Patients undergoing surgical treatment of the aortic arch (DE) were further subdivided according to the extent of resection and reimplantation of the epi-aortic vessels. In-hospital mortality in patients undergoing arch concavity replacement (hemi-arch resection) was 31.5%, while in patients undergoing arch replacement with epi-aortic trunks’ reimplantation with or without extension to the descending aorta by Frozen Elephant Trunk was 52.5%, with a *p*-value 0.022. Regarding late results of these patients, in the hemi-arch vs. total arch replacement, the survival at 92 months was 61 ± 9% vs. 67 ± 19% (*p* = 0.539) (Figure 5A), freedom from re-intervention 70 ± 9% vs. 85 ± 10% (*p* = 0.55) (Figure 5B) and freedom from re-intervention and death 46 ± 9% vs. 57 ± 18% (*p* = 0.36) (Figure 5C). The distal re-intervention rate was 9.5% in the hemi-arch group and 10.5% in the total arch replacement group. No patients undergoing combined aortic root and total arch replacement underwent re-intervention. No independent predictors of reduced freedom from aorta-related events or risk factors for reduced survival were identified in the statistical analyses.

## 4. Discussion

Acute aortic dissection is a life-threatening condition and a real challenge for cardiac surgeons. The mortality rate for untreated dissections is 50% in the first 48 h and 75% within two weeks of the onset of the event [10,11]. Despite numerous innovations in diagnosis and medical and surgical treatment, in-hospital mortality remains high, ranging from 15 to 30%. The choice of surgical strategy plays a crucial role in the management of these patients, since it is well-known that the postoperative course can be influenced by the type of surgical procedure used, as well as, of course, by the patient’s clinical picture and preoperative status [12,13,14,15,16]. There is still no surgical consensus on the best management of proximal and distal aortic segments in the treatment of AAD. The standard treatment in patients with AAD is surgery of the proximal aorta, with replacement of the ascending thoracic aorta including the intimal breach area, possibly combined with aortic valve treatment if insufficient. However, some surgeons advocate a more aggressive approach, extending treatment to the aortic arch and root in an attempt to reduce the risk of future dilatation but running the risk of a higher operative mortality rate. On the other hand, the inspection of the aortic arch is vital to identify any intimal or re-entry lesions; between 10 and 30% of AAD cases have an intimal arch lesion [17], while a large proportion of patients have an aortic arch dissection, even in the absence of local intimal lesions. In the latter group of patients, several surgeons advocate a more conservative “tear oriented” approach with arch concavity replacement to reduce mortality and postoperative morbidity [18,19,20], while others advocate a more aggressive approach with total aortic arch replacement with or without the use of the “Frozen Elephant Trunk” or the classic, or a modified “Elephant Trunk technique” [12,21,22]. 

There are considerable difficulties in determining the best practice, and it is difficult to assess the appropriateness and outcome of surgery by constructing groups of patients with similar preoperative characteristics, as many factors determine the aggressiveness and complexity of the operations. In our study, we reported 10 years of experience in the management of 269 patients undergoing surgery for AAD, subdividing the patients according to the type of the received surgical treatment. The choice of surgical technique depended on several factors, i.e., aortic root and arch diameter, patient age, clinical presentation, Marfan syndrome and aortic valve dysfunction. Without accurate analyses of these data, it is not possible to identify the most appropriate surgical treatment. Nonetheless, according to our experience, an aggressive proximal approach, including aortic root and valve replacement with reimplantation of the coronary ostia, was an excellent treatment strategy, with an operative mortality of 19% for the Bentall operation not combined with a distal treatment. In our study, the aortic arch was explored in all patients, and the choice of the type of distal procedure was based on identification of the site of the intimal lesion. One hundred and thirty-two patients (49%) underwent distal extension treatment (including 20 with concomitant aortic root surgery). Complete aortic arch replacement was performed in 40 patients (four with the Elephant Trunk technique and 11 with E-Vita prosthesis). Replacement of the arch concavity alone was performed in 38 patients (4 patients with Elephant Trunk technique and 11 with E-Vita prosthesis). We replaced the arch concavity only when the lesions were found in the distal portion of the ascending aorta or in the arch concavity, or according to the surgeon’s preference. The main aim of surgical treatment is to achieve the obliteration of the false lumen and subsequent complete thrombosis. Unfortunately, it is well-known that even extensive treatment often fails to achieve this goal. The re-intervention rate varies between 15 and 30% at 10 years, although in most studies in the literature, it is not clear whether the dilatation involves the aortic root or the distal aorta [23]. In our study, the total incidence of redo operation was 16.9%, with a distal re-intervention rate of about 10% whatever the surgical treatment performed, regardless of its extension into the aortic arch or not. The number of patients who underwent an aortic root re-intervention was zero if they received proximal radical treatment, and significantly lower if they received an extended aortic arch treatment in comparison with patients undergoing isolated ascending aortic replacement. While the reason for the absence of proximal re-interventions in patients undergoing radical aortic root surgery seems obvious, it is less clear why arch extension also indirectly reduced the risk of root re-interventions. In our test population, the overall probability of freedom from a re-intervention was 71 ± 5% at 92 months and was higher in patients undergoing aortic root replacement, although not reaching statistical significance. Patients who underwent aortic arch treatment showed a reduced late survival. In fact, compared with patients who did not undergo distal extension treatment, these patients had a freedom from re-intervention of 73 ± 7.5% vs. 70.5 ± 6.5% (*p* = 0.8), respectively, and the probability of freedom from re-intervention and death was 48 ± 8% vs. 53 ± 7% (*p* = 0.95). Fattori and co-workers reported, in a cohort of seventy patients, a descending aortic growth rate of 3.7 mm/year in patients with a pervious false lumen, a rate significantly higher than in patients without a pervious false lumen (only 1.1 mm/year) [24]. Aortic expansion is more common and more rapid at the level of the descending tract, as demonstrated by Zierer, who identified aortic diameter, blood pressure and patency of the false lumen as independent predictors [25]. In the case of complete aortic arch replacement with the reimplantation of epi-aortic trunks, adequate and effective cerebral, myocardial and splanchnic protection methods must be ensured.

Permanent neurological damage remains one of the most significant and disabling complications of surgery. Its incidence varies between 5.5 and 33.3%, depending on the type of treatment performed, patient age, preoperative conditions and cerebral perfusion strategies. Several techniques have been developed to improve the safety and efficacy of cerebral protection during aortic arch surgery. However, the optimal modality is still debated. Theoretically, in surgeries requiring longer ischemic times, bilateral anterograde selective cerebral perfusion combined with moderate hypothermia, according to the Kazui technique, would seem to be the best option rather than unilateral cerebral perfusion. In fact, we observed no significant difference between the two perfusion strategies used; the overall rate of early permanent neurological damage was 12.3% (10.9% in the case of unilateral perfusion vs. 15.8% in the case of bilateral perfusion, *p* = 0.45). The choice of the best site for arterial cannulation is also a matter of debate. Femoral cannulation has been identified as an independent predictor of hospital mortality and associated with a worse neurological outcome, probably due to the risk of retrograde cerebral embolization. Femoral cannulation may indeed lead to selective retrograde perfusion of the false lumen, causing cerebral or visceral malperfusion [16]. It may also lead to the dislocation of atherosclerotic plaques, resulting in peripheral or cerebral damage [26,27]. On the other hand, the axillary artery seems to be burdened with a lower incidence of atherosclerosis and therefore less likely to be dissected. In our center, cannulation of the femoral or axillary artery, which was used both for cerebral perfusion by the Kazui technique and by partial tangential clamping of the upper part of the aortic arch, did not show significant differences in terms of in-hospital mortality or permanent neurological damage. Several authors suggest, in agreement with IRAD (International Registry of Aortic Dissection) data, that early survival is related more to preoperative complications and patient comorbidities than to the type of surgical management [1,2,3,4,5]. 

The main independent preoperative predictors of mortality identified in the IRAD registry were previous aortic valve replacement, migrating pain, preoperative limb ischemia, hypotension, shock and cardiac tamponade. In our study, although prolonged ventilation, postoperative acute renal failure, limb ischemia, concomitant aortic valve replacement, extension to the aortic arch, clamping time and circulatory arrest had a strong impact in univariate analysis, only age (*p* = 0.009), cardiopulmonary bypass time (*p* = 0.006) and preoperative orotracheal intubation (*p* = 0.022) were independently predictive of in-hospital mortality. In particular, in our case series, patients aged > 75 years had significantly higher mortality than younger patients did (52 vs. 27%, *p* = 0.018). Unfortunately, two of the three factors that significantly affected operative mortality are not modifiable, and the prognosis seems to be closely related to patient characteristics, comorbidities and dissection-related complications rather than to the technical choice made, in agreement with what has been reported in the IRAD registry by Berretta and colleagues in their analysis of predictors of hospital mortality [28]. Data from the IRAD show that about 1/3 of patients diagnosed with aortic dissection are over 70 years of age. Only 47.6% of those over 80 years of age with AAD underwent surgery [29]. Although surgical mortality increases significantly with increasing age (38.2% in patients > 70 years compared with 26% in younger patients), surgical management is still associated with significantly lower in-hospital mortality compared with medical treatment alone (23.8 vs. 59.3%, in patients < 80 years). A surgical approach should therefore be considered and preferred in all patients with an acute type A acute aortic dissection and should not be denied solely on the basis of age, unless, of course, there are severe and disabling associated diseases. Above 80 years, however, medical therapy may be a viable alternative. According to the results of our case series, treatment in such patients should be aimed at survival rather than radical surgical treatment. Therefore, in view of the reduced life expectancy, it would be appropriate to limit the extent of replacement to the ascending aorta, even in the presence of a dissecting or aneurysmal process in the aortic root or arch, as suggested by Komatsu and colleagues [30]. Age is therefore a risk factor for mortality in patients with aortic dissection, although it is not in itself an absolute contraindication to surgical treatment. In a recent paper published by another Italian group, the introduction of hybrid prostheses allowed for a significant decrease in early mortality rate, probably due to a more radical resection [31]. Hybrid prosthesis can often be useful in decreasing operation difficulty, and are actually believed to induce better long-term results, mainly in the distal aortic tract. The combination of aggressive root and arch treatment might change significantly the long-term destiny of these patients. Analysis of aortic dimensions in our study showed that the maximum transverse diameter at the time of dissection of 54.2 ± 10.4 mm; this value, although distorted by acute expansion during dissection, is well below the target identified by the guidelines for the treatment of ascending thoracic aortic aneurysms. Thus, the initial aortic diameter does not appear to be the sole determinant of the dissection process, even in the absence of bicuspid or associated connective tissue disease. In fact, about 50% of patients undergo dissection at aortic diameters smaller than those indicated for the elective treatment of expansive aortic aneurysms [32,33].

### Study Limitations

The fate of the false lumen was not investigated, so we had no data on its patency in the overall population, nor in patients undergoing anticoagulant therapy for atrial fibrillation or mechanical valve placement. Consequently, we were unable to clarify the role of persistence of the false lumen in affecting late prognosis in terms of survival and re-intervention rate. There were no reliable data on the real necessity of preoperative intubation; in an unknown percentage of patients, it was probably performed prophylactically for safer transport to hospital, affecting the correct assessment of the preoperative neurological picture and clinical conditions. History taking was often difficult and incomplete due to the emergency nature of the treatment. Even the indirect history that could be obtained by relatives was often not possible due to discrepancies in arrival times between patients and relatives. No valve-sparing surgery was performed in the study population. Although such interventions are theoretically ideal in the treatment of AAD, as they would eliminate the aortic root while preserving the very-often-healthy native valve, they require long cardiopulmonary bypass and cross-clamp times, along with a meticulous surgical technique. The highly variable anatomy and the fragility of the structures therefore make the most complex procedures very risky. In addition, the results may also be affected by the variability of techniques and strategies adopted, and by the experience and personal beliefs of the operating surgeon. Finally, the adoption of hybrid prostheses was not evaluated on purpose: its diffuse use might be able to prevent a considerable number of distal aorta complications. Indeed, our data underline that a radical treatment of the aortic root could be of utmost importance in preventing the most life-threatening complications and therefore improving the patients’ long-term prognosis.

## 5. Conclusions

In the treatment of the type A of acute aortic dissection, all adopted surgical strategies were associated with satisfactory long-term survival. In the group of patients in which the aortic root had not been replaced, we observed significantly reduced event-free survival. According to our experience, an aggressive proximal approach, including aortic root and valve replacement with reimplantation of the coronary ostia, was an excellent treatment strategy, with an in-hospital mortality rate of 19% for Bentall surgery not combined with distal treatment.

## Figures and Tables

**Table 1:** Preoperative characteristics in the general population.

Variable	
Age (years), mean ± SD	63.1 ± 12.8
Age > 75 years, *n* (%)	48 (17)
Female, *n* (%)	78 (29)
BMI (kg/m^2^), mean ± SD	27.5 ± 5.1
BSA (m^2^), mean ± SD	1.96 ± 0.23
Hypertension, *n* (%)	238 (88.5)
Family history, *n* (%)	29 (10.8)
Diabetes, *n* (%)	17 (6.3)
Tobacco use, *n* (%)	76 (28.3)
Obesity, *n* (%)	63 (23.4)
Coronary artery disease, *n* (%)	25 (9.3)
Previous cardiac surgery, *n* (%)	17 (6.3)
Preoperative IOT, *n* (%)	40 (14.9)
Preoperative neurological damage, *n* (%)	35 (13)
Diameter of the ascending aorta (mm), mean ± SD	54.2 ± 10.4

**Table 2:** Preoperative variables divided into groups of patients.

Variable	No Extension (NE) (*n* = 96)	Proximal Extension (PE) (*n* = 41)	Distal Extension (DE) (*n* = 112)	Bilateral Extension (BE) (*n* = 20)	*p*-Value
Age (years), mean ± SD	65.2 ± 11.9	55.3 ± 14.9	65.1 ± 10.9	57.4 ± 14.6	0.0001
Age > 75 years, *n* (%)	22 (23)	2 (5)	24 (21)	0 (0)	0.0001
Female, *n* (%)	38 (39.6)	8 (19.5)	29 (25.9)	3 (15)	0.024
BMI (kg/m^2^), mean ± SD	26.7 ± 4.5	27.2 ± 4.5	28.3 ± 5.7	28.3 ± 4.2	0.114
BSA (m^2^), mean ± SD	1.89 ± 0.20	2.0 ± 0.25	1.99 ± 0.24	2.02 ± 0.18	0.002
Hypertension, *n* (%)	86 (89.6)	35 (84.4)	99 (88.4)	18 (90)	0.908
Family history, *n* (%)	14 (14.6)	5 (12.2)	9 (8)	1 (5)	0.376
Diabetes, *n* (%)	7 (7.3)	2 (4.9)	6 (5.3)	2 (10)	0.818
Tobacco use, *n* (%)	30 (31.2)	13 (31.7)	28 (25)	5 (25)	0.716
Obesity, *n* (%)	16 (16.7)	11 (26.8)	30 (26.8)	6 (30)	0.244
Coronary artery disease, *n* (%)	8 (8.3)	4 (9.7)	9 (8)	4 (20)	0.385
Previous cardiac surgery, *n* (%)	5 (5.2)	5 (12.2)	7 (6.2)	0 (0)	0.268
Preoperative IOT, *n* (%)	11 (11.5)	8 (19.5)	18 (16.1)	3 (15)	0.634
Preoperative neurological damage, *n* (%)	10 (10.4)	4 (9.8)	16 (14.3)	5 (25)	0.314

**Table 3:** Preoperative variables in the ED group.

Variable	Hemi-Arch (*n* = 92)	Aortic Arch (*n* = 40)	*p*-Value
Age (years), mean ± SD	65.5 ± 10.9	60.2 ± 13.1	0.016
Age > 75 years, *n* (%)	19 (20)	5 (12)	0.246
Female, *n* (%)	24 (26.1)	8 (20)	0.453
BMI (kg/m^2^), mean ± SD	28.2 ± 5.8	28.5 ± 4.8	0.768
BSA (m^2^), mean ± SD	1.99 ± 0.2	2.00 ± 0.2	0.806
Hypertension, *n* (%)	78 (84.8)	38 (95)	0.129
Family history, *n* (%)	7 (7.6)	3 (7.5)	0.983
Diabetes, *n* (%)	5 (5.4)	3 (7.5)	0.648
Tobacco use, *n* (%)	22 (23.9)	11 (27.5)	0.662
Obesity, *n* (%)	23 (25)	13 (32.5)	0.414
Coronary artery disease, *n* (%)	10 (10.9)	3 (7.5)	0.550
Previous cardiac surgery, *n* (%)	6 (6.5)	1 (2.5)	0.343
Preoperative OTI, *n* (%)	12 (13)	9(22.5)	0.169
Preoperative neurological damage, *n* (%)	14 (15.2)	7 (17.5)	0.716

ED = distal extension; OTI = orotracheal intubation.

**Table 4:** Intraoperative variables in the general population.

Variable	
Axillary cannulation, *n* (%)	134 (49.8)
Femoral cannulation, *n* (%)	112 (41.6)
Central cannulation, *n* (%)	23 (8.6)
Monolateral perfusion, *n* (%)	82 (30.5)
Bilateral perfusion, *n* (%)	80 (29.7)
CPB time (min), *n* (%)	169 ± 84.8
X-Clamp time (min), mean ± SD	97 ± 49.4
Circulatory arrest time (min), mean ± SD	39 ± 31.6
Circulatory arrest temperature (°C), mean ± SD	27 ± 2.4
Concomitant procedures:	
Aortic valve replacement, *n* (%)	17 (6.3)
CABG, *n* (%)	22 (8.2)

**Table 5:** Intraoperative variables divided for groups of patients.

Variable	No Extension (*n* = 96)	Proximal Extension (*n* = 41)	Distal Extension (*n* = 112)	Bilateral Extension (*n* = 20)	*p*-Value
Axillary cannulation, *n* (%)	25 (26.0)	17 (41.5)	78 (69.6)	14 (70.0)	0.0001
Femoral cannulation, *n* (%)	56 (58.3)	20 (48.8)	30 (26.8)	6 (30.0)	0.0001
Central cannulation, *n* (%)	15 (15.6)	4 (9.7)	4 (3.6)	0 (0)	0.009
Monolateral perfusion, *n* (%)	14 (14.6)	8 (19.5)	50 (44.6)	10 (50.0)	0.0001
Bilateral perfusion, *n* (%)	5 (5.2)	3 (7.3)	62 (55.3)	10 (50.0)	0.0001
CPB time (min), *n* (%)	107 ± 53	195 ± 91.2	196 ± 74.4	250 ± 76.1	0.0001
X-Clamp time (min), mean ± SD	65 ± 30.1	127 ± 43.0	102 ± 49.3	151 ± 43.1	0.0001
Circulatory arrest time (min), mean ± SD	17 ± 17.6	22 ± 16.3	46 ± 33.6	37 ± 24.4	0.0001
Circulatory arrest temperature (°C), mean ± SD	28 ± 3.0	27 ± 3.1	27 ± 2.1	26 ± 1.9	0.038
Concomitant procedures:					
Aortic valve replacement, *n* (%)	11 (10.4)	0 (0)	6 (53.6)	0 (0)	0.036
CABG, *n* (%)	4 (4.2)	8 (19.5)	7 (6.2)	3 (15.0)	0.012

**Table 6:** Intraoperative variables in DE group.

Variable	Hemi-Arch (*n* = 92)	Aortic Arch (*n* = 40)	*p*-Value
Axillary Cannulation, *n* (%)	63 (68.5)	29 (72.5)	0.644
Femoral Cannulation, *n* (%)	25 (27.2)	11 (27.5)	0.969
Central Cannulation, *n* (%)	4 (4.3)	0 (0)	0.180
Monolateral Perfusion, *n* (%)	39 (42.4)	21 (52.5)	0.284
Bilateral Perfusion, *n* (%)	53 (57.6)	19 (47.5)	0.284
CPB time (min), *n* (%)	183 ± 64.2	254 ± 80.9	0.0001
X-Clamp time (min), mean ± SD	94 ± 40.7	146 ± 55.5	0.0001
Circulatory Arrest Time (min), mean ± SD	32 ± 19.6	76 ± 36.3	0.0001
Concomitant procedures:			
Aortic valve replacement, *n* (%)	5 (5.4)	1 (2.5)	0.457
CABG, *n* (%)	8 (8.7)	2 (5.0)	0.461

**Table 7:** Intraoperative variables in the DE group.

Variable	
Mortality, *n* (%):	86 (32.0)
Intraoperative, *n* (%)	23 (26.7)
Postoperative, *n* (%)	63 (73.3)
Postoperative causes of mortality:	
MOF, *n* (%)	29 (10.8)
LCOS, *n* (%)	12 (4.5)
Septic shock, *n* (%)	11 (4.1)
Coma, *n* (%)	2 (0.7)
Other, *n* (%)	9 (3.3)
Permanent neurological damage, *n* (%)	28 (10.4)
Transitory neurological damage, *n* (%)	17 (6.3)
Organ ischemia, *n* (%)	7 (2.6)
Respiratory failure, *n* (%)	75 (27.9)
Acute kidney Injury, *n* (%)	49 (18.2)

MOF = multiple organ failure; LCOS = low cardiac output syndrome.

**Table 8:** Postoperative variables divided according to groups of patients.

Variable	No Extension (*n* = 96)	Proximal Extension (*n* = 41)	Distal Extension (*n* = 112)	Bilateral Extension (*n* = 20)	*p*-Value
Mortality, *n* (%)	28 (29.1)	8 (19.5)	43 (38.4)	7 (35.0)	0.140
Intraoperative, *n* (%)	5 (17.9)	2 (25)	12 (27.9)	4 (20.0)	0.089
Postoperative, *n* (%)	23 (82.1)	6 (75)	31 (72.1)	3 (80)	0.254
Postoperative causes of mortality:					
MOF, *n* (%)	11 (11.5)	2 (4.9)	14 (12.5)	2 (10.0)	0.542
LCO, *n* (%)	6 (6.2)	2 (4.9)	4 (3.6)	0 (0)	0.666
Septic Shock, *n* (%)	3 (3.1)	2 (4.9)	6 (5.4)	0 (0)	0.653
Coma, *n* (%)	1 (1.0)	0 (0)	1 (0.9)	0 (0)	0.899
Other, n (%)	2 (2.1)	0 (0)	6 (5.4)	1 (5.0)	0.273
Permanent neurological injury, *n* (%)	13 (13.5)	3 (7.3)	10 (8.9)	2 (10.0)	0.660
Transitory neurological injury, *n* (%)	5 (5.2)	2 (4.9)	10 (8.9)	0 (0)	0.388
Organ ischemia, *n* (%)	2 (2.1)	2 (4.9)	3 (2.7)	0 (0)	0.729
Respiratory failure, *n* (%)	30 (31.2)	9 (21.9)	32 (28.6)	4 (20.0)	0.607
Acute kidney Injury, *n* (%)	17 (17.7)	4 (9.8)	24 (21.4)	4 (20.0)	0.274

**Table 9:** Postoperative variables in the ED group.

Variable	Emiarch (*n* = 92)	Aortic Arch (*n* = 40)	*p*-Value
Mortality, *n* (%)	29 (31.5)	21 (52.5)	0.022
Intraoperative, *n* (%)	7 (24.1)	9 (42.9)	0.013
Postoperative, *n* (%)	22 (75.9)	12 (57.1)	0.179
Postoperative causes of mortality:			
MOF, *n* (%)	13 (14.1)	3 (7.5)	0.438
LCO, *n* (%)	3 (3.3)	1 (2.5)	0.937
Septic Shock, *n* (%)	4 (4.3)	2 (5)	0.707
Coma, *n* (%)	1 (1.1)	0 (0)	0.544
Other, *n* (%)	1 (1.1)	6 (15)	0.000
Permanent neurological injury, *n* (%)	11 (12)	1 (2.5)	0.139
Transitory neurological injury, *n* (%)	7 (7.6)	3 (7.5)	0.768
Organ ischemia, *n* (%)	2 (2.2)	1 (2.5)	0.772
Respiratory failure, *n* (%)	28 (30.4)	8 (20)	0.500
Acute kidney Injury, *n* (%)	23 (25)	5 (12.5)	0.242

**Table 10:** Independent predictors of in-hospital mortality.

Variable	Odds Ratio	CI 95%	*p*-Value
Age ^a^	1.04	1.01—1.08	0.009
CPB ^a^	1.01	1.003—1.02	0.006
Preoperative OTI	3.40	1.19—9.64	0.022

^a^ increasing values; CPB = cardiopulmonary bypass time; CI = confidence interval.

**Table 11:** Actual rate of re-intervention.

Groups	
General population	16.9%
NE	20.3%
PE	8.7%
DE	12.6%
BE	0%

NE = no extension, PE = proximal extension, DE = distal extension, BE = bilateral extension.

## Data Availability

In-hospital database.
